# Inflammation mediated Osteoclastogenesis and bone resorption markers in Periodontitis subjects with and without type 2 diabetes- A Cross-sectional study

**DOI:** 10.1016/j.jobcr.2026.101484

**Published:** 2026-07-02

**Authors:** Ramalingam Mathangi, Ranganathan Parthasarathy, Nalini Devarajan, S. Bhuminathan, BagavadGita Jayaraman

**Affiliations:** a Department of Biochemistry, Sree Balaji Dental College and Hospital, Bharath Institute of Higher Education and Research, Chennai, Tamil Nadu, India; b Departments of Psychiatry, Medicine, Neuroscience and Biochemistry, University of Louisville, Louisville, KY, 40202, USA; c Central Research Laboratory, Meenakshi Institute of Higher Education and Research (MAHER), Chennai, Tamil Nādu, India; d Department of Prosthodontics, Sree Balaji Dental College and Hospital, Bharath Institute of Higher Education and Research, Chennai, Tamil Nadu, India; e Chennai Dental Research Foundation, Chennai, India

**Keywords:** Cathepsin K, Periodontitis, osteoclastogenesis, Diabetes mellitus,type2, Bone resorption, Alveolar boneloss, RANK ligand, Interleukin-6

## Abstract

**Objectives:**

Periodontitis and type2 diabetes are chronic inflammatory diseases resulting in osteoclastogenesis and bone loss**.** This study investigated the effect of type2 diabetes in inflammation mediated osteoclastogenesis invitro along with bone resorption markers in subjects with and without periodontitis.

**Methods:**

Whole blood samples were collected from periodontitis subjects with (n = 30) or without T2D (n = 30) and healthy subjects (n = 30). Isolated PBMCs were cultured to study osteoclastogenesis pathways *invitro*. The prime markers of RANKL signalling pathway, including TRAF6, cFos, NFATc1 and cathepsin K, were analysed by immunoblotting. Serum interleukin 6 (IL6), C- terminal telopeptide of Type I collagen (CTx), tartrate resistant acid phosphatase 5b (TRAP5b) and alkaline phosphatase (ALP) were estimated in all groups.

**Results:**

Our results showed that cultured PBMCs from periodontitis and T2D subjects demonstrated a higher number of TRAP positive cells compared to subjects without T2D. IL6, CTx and TRAP5b levels were significantly elevated in T2D with periodontitis, compared to normal subjects with or without periodontitis.

**Conclusion:**

This study provides insights on inflammation mediated bone remodelling T2D in periodontitis leading to alveolar bone loss. Understanding this molecular mechanism might provide future translational potential and therapeutic interventions for the osteoclast-related pathological bone diseases such as chronic periodontitis, enabling prevention of tooth loss.

## Abbreviations

T2D: Type2 Diabetes
RANKL -: Receptor activator of NFκB ligand
TRAF6 -: Tumour Necrosis factor receptor associated factor 6
NSAIDs -: Non steroidal anti inflamamtory drugs
NFATc1: Nuclear Factor of Activated T Cell 1
PBMC -: Peripheral Blood Mononuclear Cells
IL6-: Interleukin- 6
ELISA –: Enzyme Linked Immuno Sorbent Assay
CTx -: C- Terminal Telopeptide of Type I Collagen
TRAP5b -: Tartrate Resistant Acid Phosphatase 5b
GCF: Gingival Crevicular Fluid
HbA1c –: Glycated Haemoglobin
ALP -: Alkaline Phosphatase
CAL-: Clinical Attachment Level
BOP -: Bleeding on Probing
PPD -: Periodontal Probing Depth
TRAP: Tartrate Resistant Acid Phosphatase

## 1 Introduction

Periodontitis is a chronic inflammatory disorder that affects dental health and specifically destroys the supporting structures leading, to premature loss of teeth. With a prevalence of 11.2%, periodontitis affects 743 million people across the globe.^1^ The primary etiological cause of periodontitis is the host inflammatory response to key periodontal pathogens such as *P. gingivalis, T. forsythia* and *A. actinomeycetemcomitans*.The chronic inflammation resulting from periodontitis leads to exacerbated loss of the tooth-supporting connective tissue, including gingiva, periodontal ligament and alveolar bone. This vicious cycle of dysregulated inflammatory responses results in bone loss, a radiographic diagnostic indicator of periodontitis.^2^ Type2 Diabetes (T2D) plays a pivotal role in enhancing systemic inflammation as well as bone loss^3^ and it links several physiological and biochemical mechanisms that lead to periodontitis.^4^

Edentulism, due to periodontitis, is a major concern, and patients suffering from T2D have a three - fold risk in bone loss compared to people not affected from diabetes.^5^ More specifically, pro-inflammatory cytokine IL-6 mediates osteoclastogenesis, a process upregulating receptor activator of NFκB ligand (RANKL), which in turn activates a cascade of pathways involving Tumour Necrosis factor receptor associated factor 6 (TRAF6), cFos, a proto-oncogene, and nuclear factor of activated T cell1(NFATc1). All these pathways lead to abnormal bone resorption.^6^ In T2D, there is an elevated inflammatory response adding systemically to the pre-existing inflammation in the periodontium which markedly activates osteoclast differentiation and bone loss. Periodontitis induced alveolar bone loss is identified as a co-morbidity of T2D.^7^ The significant role of sustained hyperglycemia of T2D is to induce osteoclastogenesis and bone resorption especially in periodontitis and this mechanism is poorly understood at molecular level.

Diabetes increases susceptibility to destructive periodontal diseases. Studies in both in humans and animals have shown that these two disorders enhance inflammation in the periodontium and severely increase the risk of periodontitis. For every unit increase in HbA1c levels, the risk of periodontitis increases by 64%.^8^ Conversely, periodontal therapy can significantly reduce HbA1c levels decreasing the load of systemic inflammation.^9^ Furthermore, RANKL mediated osteoclastogenesis and diminished bone regeneration have been reported in diabetes.^10^ The upregulation of inflammatory cytokines and bone metabolism in advanced glycation is studied in mouse osteocytes.^11^ Also, hyperglycemia influenced oxidative telomeric changes in periodontal ligament stem cells (PDLSC) enhancing bone loss is studied in detail in mouse model.^12^

Although the bidirectional relationship between periodontitis and type2 diabetes mellitus (T2D) is well established,^13^ the molecular mechanisms by which diabetes-associated hyperglycemia directly enhances osteoclastogenesis and alveolar bone loss remain poorly understood. Current evidence suggests that diabetes exacerbates periodontal inflammation and bone resorption mainly through RANKL-mediated pathways; however, most mechanistic insights are derived from animal models or clinical biomarker studies rather than human cell-based systems.^14^ There is limited direct evidence defining alterations in downstream RANKL signalling molecules such as TRAF6, cFos, and NFATc1 in human osteoclast precursors under diabetic conditions.

Therefore, the present study was designed to explore how type2 diabetes–associated hyperglycemia influences RANKL-induced osteoclast formation using an in vitro PBMC differentiation model. The progression of osteoclastogenesis was examined by assessing the expression of key signalling proteins involved in osteoclast differentiation, including TRAF6, cFos, NFATc1, and CathepsinK. In parallel, circulating markers of bone resorption (TRAP5b and CTx), bone formation (alkaline phosphatase), and systemic inflammation (IL-6) were evaluated to better understand the interplay between diabetes-related inflammation and altered bone turnover in periodontitis.

## 2 Methods

### 2.1 Study design and participants

This study is designed as a cross-sectional study and was approved by the institutional ethical committee (SBDC - ECM 104/01/15/04). Subjects in the age group between 45 and 60 years those seeking dental care from the Department of Periodontology, from a private dental college were recruited for this study and written informed consents were obtained according to the Indian Council of Medical Research (ICMR) and World Medical Association Declaration of Helsinki Guidelines. This study was carried out in accordance with the Strengthening the Reporting of OBservational studies in Epidemiology (STROBE) guidelines.

The sample size was calculated using formula N = Z^2^P(1-P)/d^2^. With the power of 90% and alpha value of 0.05 the sample size obtained was 27 per group. To ensure that each group had an equal number of patients and to account for potential errors in sample collection and handling, the minimum number of patients needed was raised to 90, or 30 patients per group.

A total of 90 subjects were included in this study and categorized into three groups.

1. Group I (n = 30) – Healthy controls (HC).
2. Group II (n = 30) – Subjects with periodontitis (PD).
3. Group III (n = 30) - Subjects with T2D and periodontitis (T2D + PD).

### 2.2 Inclusion and exclusion criteria

Selection of periodontitis subjects was based on European Federation of Periodontology/American Academy of Periodontology (EFP/AAP) case classification of 2017.^15^ Patients in group II (PD) fall under stage III and Grade B and Patients under group III (T2D + PD) fall under stage III and IV and grade B. While healthy controls are classified under stage I and Grade A. Patients with >20 teeth were considered for the study. Subjects with Periodontal probing Depth (PPD) ≥ 6 mm detectable at ≥2 non-adjacent sites were included. Hyperglycemia was confirmed by measuring Fasting Blood Sugar (FBS), post prandial (PP) and HbA1c levels. Subjects with fasting blood sugar levels ≥126 mg/dl, post prandial >200 mg/dl and HbA1c ≥ 7% were considered diabetic.^16^

Pregnant women, smokers, and subjects on bone remodelling drugs such as bisphosphonates, NSAIDs, antibiotic therapy for the past three months or any other periodontal therapy in the past six months were excluded. Subjects with other systemic diseases which could influence progression of periodontitis were also excluded. Information related to medical and dental history was obtained through a well-organized questionnaire [https://www.periocafe.com/wp-content/uploads/Medical-Dental-Questionnaire.pdf] and stored in an anonymous database with security for HIPPA compliance.

### 2.3 Screening for periodontitis

A trained examiner assessed the clinical periodontal parameters and subjects were given a clinical periodontal examination to ascertain Periodontal Probing Depth (PPD), Bleeding on Probing (BOP) and Clinical attachment level (CAL) for each tooth. Measurements were performed using UNC-15 probe. Assessment of plaque was also carried out using the modified Löe and Silness gingival index.^17^ Full mouth probing was done to determine CAL and PPD values. Six periodontal sites were measured per tooth. Interdental CAL ≥5 mm or Buccal or oral CAL ≥3 mm detectable at ≥2 non-adjacent teeth were used to assess severity.

### 2.4 Isolation of Peripheral Blood Mononuclear Cells (PBMCs) from whole blood

Seven ml of whole blood was diluted in 1:2 with phosphate buffered saline (PBS). Two parts of diluted blood were layered on top of one-part Ficoll-Paque medium (Sigma Aldrich Co., USA) and centrifuged at 1700 X *g* for 30 min at room temperature (RT). The cell layer on top of the Ficoll was collected, resuspended in 1X PBS, and centrifuged at 1700 X *g* for 10 min three times to obtain pure cells. Subsequently, cells were counted in a hemacytometer after trypan blue staining.

### 2.5 Generation and differentiation of human primary PBMCs

Isolated peripheral blood mononuclear cells (PBMCs) were cultured in 24 well plates (1 × 10^6^ cells per well) in Dulbecco's modified Eagle medium (DMEM) containing 10% FBS, with Macrophage Colony stimulating factor (M-CSF- 40 ng/ml), soluble receptor activator of NFκB ligand (sRANKL- 25 ng/ml) for 21 days. Cultures were fed every 3–4 days with fresh medium. All chemicals used were of research and analytical grades and were obtained from Sigma-Aldrich, Inc (St. Louis, MO, USA).

### 2.6 Tartrate Resistant Acid Phosphatase (TRAP) staining

After cultivation for 21 days, tartrate-resistant acid phosphatase (TRAP) assay was carried out.^18^ TRAP positive (TRAP positive) multinucleated cells with more than three nuclei were identified as mature osteoclasts and counted as osteoclasts with an inverted phase contrast microscope.^19^ The TRAP assay was carried out in triplicates.

### 2.7 Quantification of osteoclast precursors

To determine the percentage of CD14-positive (CD14^+^) cells, flow cytometry was performed. PBMCs (1 × 10^6^) were centrifuged at 1700 × g for 10 min and incubated in PBS with a 1:10 dilution of FITC-conjugated anti-human CD14 antibody for 30 min. After staining, the cells were washed, resuspended in 1× PBS, and analysed by flow cytometry.

### 2.8 Western blotting

PBMCs after 21 days of culture with RANKL and M-CSF were isolated by brief centrifugation. Cells were lysed in radio-immuno precipitation assay buffer (RIPA) and lysates were centrifuged at 10,000 X *g* at 4°C for 20 min. The supernatant was analysed for protein content using the BCA method (BCA protein assay kit, Thermo-Fisher, USA). Samples were denatured in a sample buffer by heating at 95°C for 5 min and then separated on 10% Sodium dodecyl sulphate polyacrylamide gel electrophoresis (SDS-PAGE). Proteins from gels were electro-transferred to polyvinylidene difluoride (PVDF) membrane. Membranes were blocked for 2h at room temperature with Tris-Buffered Saline (TBS, 50mM Tris-HCl, pH 7.5150 mM NaCl) containing 5% non-fat milk powder. The blots were washed three times with TBST (50mM Tris-HCl, pH 7.5, 150mM NaCl, and 0.02% Tween-20) and incubated with the primary antibodies against TRAF6 (1:1000 dilution), cFos (1:1000 dilution), NFATc1(1:1000 dilution), cathepsin K (CTK; 1:1000 dilution) and β-actin (1:1000 dilution) (Santacruz, TX, USA) overnight at 4°C. The blots were incubated for 1 h at room temperature with secondary antibody (1:5000 dilutions) and detected by Enhanced Chemiluminescence (ECL) detection reagent (Thermo-Scientific, USA). β-actin was used as the loading control to ensure equal amounts of sample protein were applied for comparison. Densitometric analysis was done using Image Lab ™ software (BioRad, Hercules, CA, USA).

### 2.9 Measurement of bone loss biomarkers by Enzyme Linked Immunosorbent Assay (ELISA)

Serum levels of IL6, ALP, CTx, TRAP5b were measured with ELISA kits (Shanghai Yu Biotech Co. Ltd, China) as per manufacturer's instructions.

### 2.10 Statistical analysis

All data represent experiments in triplicates. Data distribution was found to be normal using the Kolmogorov-Smirnov test. The comparison of normally distributed data among the study groups was done using One Way ANOVA followed by a Post hoc Tukey test. Statistical analysis was performed using SPSS (Version 20.0). Values are expressed as Mean ± SD and %. Values were considered significant at the levels of p < 0.01 and p < 0.001.

## 3 Results

A total of 90 subjects participated in this study. The participants were age and gender-matched to minimize potential bias.

### 3.1 Periodontal Parameters and Biochemical parameters of glycemic control

The measurements for PPD and CAL were obtained through full-mouth periodontal probing. The T2D + PD group exhibited higher BOP, PPD, and CAL values compared with the PD group. As expected, CAL was absent in the healthy controls (Table 1). Glycemic control was poor in the T2D + PD group, whereas both the PD group and the healthy controls (HCs) showed normal fasting and postprandial blood glucose levels, as well as HbA1c values. The plaque index ranged between 2 and 3 in both the PD and T2D + PD groups, with no statistically significant differences observed.

**Table 1:** Periodontal parameters across the study groups.

Periodontal parameters	Healthy controls (HC)	PD	T2D + PD
PPD (mm)	2.6 ± 1.16	5.70 ± 0.71	5.91 ± 0.61∗∗∗
CAL (mm)	0.00	5.35 ± 1.14	7.23 ± 1.50∗∗∗
BOP (%)	4.27 ± 4.73	61.01 ± 8.01	65.03 ± 13.36∗∗∗

Values represented as Mean ± SD; ∗∗∗p < 0.001; PD-periodontitis, T2D + PD -Type2 diabetes with periodontitis.

### 3.2 Effect of T2D on osteoclast differentiation with PBMC

Given that both T2D and periodontitis (PD) are associated with chronic systemic inflammation and altered bone metabolism, we hypothesized that T2D may further enhance osteoclast differentiation from peripheral blood mononuclear cells (PBMCs), thereby contributing to increased bone resorption.^20^^,^^21^

Flow cytometric analysis using an anti-human CD14^+^ antibody showed that the proportion of CD14^+^ monocyte precursors was significantly higher in the T2D + PD group compared with PD subjects and healthy controls (data not shown), indicating a greater pool of osteoclast progenitors.

**Fig. 1:**
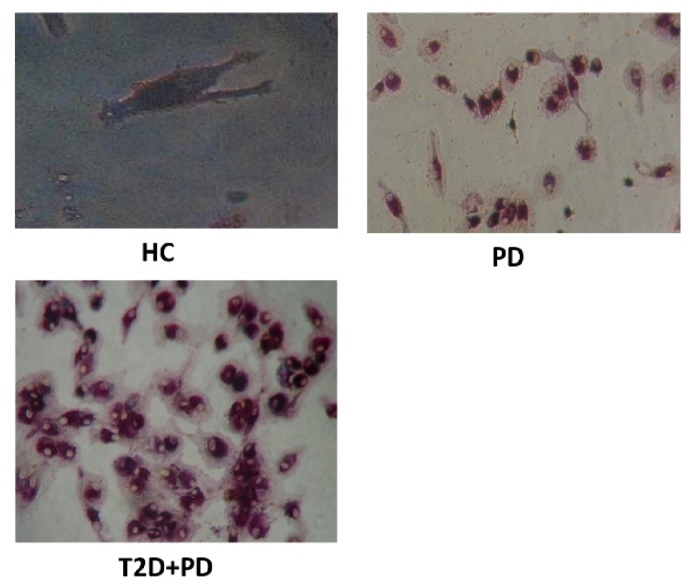

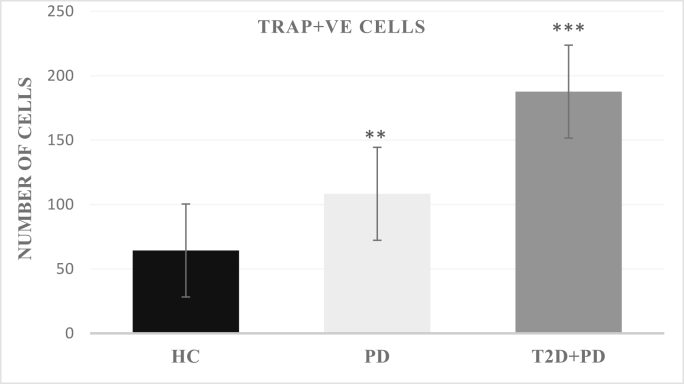
**A**. Multinucleated osteoclasts in healthy controls (HCs),PD and T2D + PD. Fig. 1B. Number of TRAP positive multinucleated osteoclasts. Significant differences were found in Periodontitis (PD) and T2D/Periodontitis (T2D + PD) conditions when compared to healthy controls (HC) (∗∗∗p < 0.001).

Microscopic observations (Fig. 1A) revealed a visibly higher number of multinucleated cells in cultures derived from T2D + PD and PD subjects compared with healthy controls, suggesting enhanced osteoclast formation under inflammatory conditions. To further confirm this, TRAP staining was used to quantify differentiated multinucleated osteoclasts per well (Fig. 1B). PBMCs obtained from T2D + PD subjects showed the highest level of osteoclast differentiation, with a mean count of 187.66 ± 8.02 cells (p < 0.001), characterized by multiple nuclei and strong TRAP positivity. In comparison, PBMC-derived osteoclasts from PD subjects were also significantly elevated (108.33 ± 7.63, p < 0.001) relative to healthy controls (64.33 ± 5.50), indicating increased osteoclastogenic potential in periodontitis alone.

Overall, these findings demonstrate that while periodontitis independently promotes osteoclastogenesis, the presence of T2D further amplifies precursor availability and osteoclast differentiation, thereby potentially accelerating inflammatory bone resorption.

### 3.3 Effect of T2D on osteoclast protein expression

**Fig. 2:**
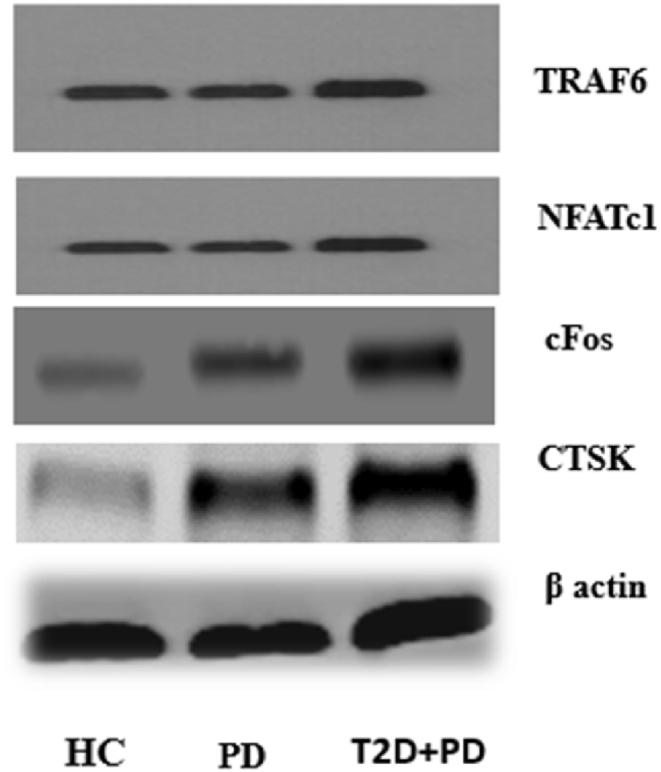

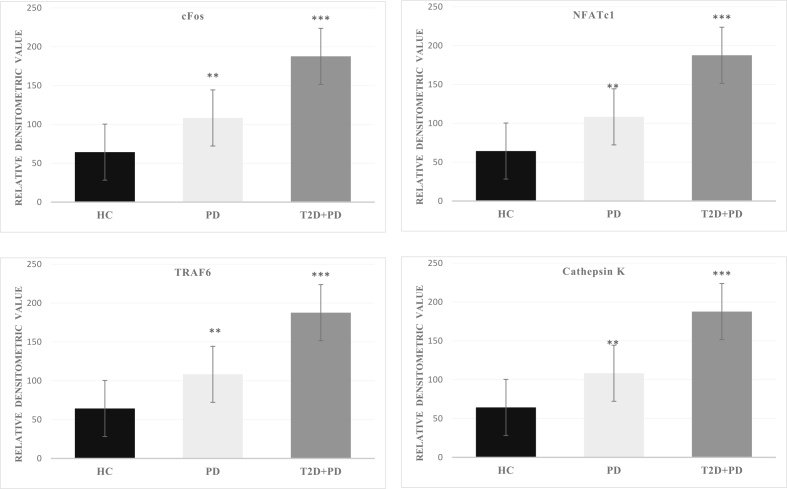
A. Western blot - Protein expression patterns of protooncogene Fos (cFos), TNF receptor associated factor 6 (TRAF6), Cathepsin K and Nuclear Factor of Activated T cell1(NFATc1) were normalised with β actin. Fig. 2B. Relative densitometric analysis in histogram. Results are expressed as mean ± SD. ∗∗Significant differences were seen between healthy controls (HC) and periodontitis (PD) (P < 0.01), ∗∗∗ significance seen between PD and T2D and periodontitis (T2D + PD) (p < 0.001).

Osteoclastic differentiation proteins, including TRAF6, cFos, NFATc1, and cathepsin K, play critical roles in osteoclast differentiation, fusion, and bone resorption. Western blot analysis was performed to assess the effect of T2D with periodontitis (T2D + PD) on the expression of selected osteoclastic markers in primed osteoclasts (Fig. 2A). A noticeable increase in the intensity and localization of these proteins was observed, particularly under the T2D + PD condition. Quantitative analysis (Fig. 2B) demonstrated that osteoclasts derived from PD subjects showed elevated expression of TRAF6 (1.5-fold), cFos (1.3-fold), NFATc1 (1.3-fold), and cathepsin K (1.4-fold) compared with healthy controls (p < 0.01). In the presence of T2D, these expression levels increased further, with TRAF6 (2.5-fold), cFos (2.0-fold), NFATc1 (2.0-fold), and cathepsin K (2.2-fold) showing greater statistical significance (p < 0.001), indicating enhanced osteoclastogenic activity.

### 3.4 Serum levels of bone resorption markers and IL-6 in T2D and Periodontitis

The serum levels of bone resorption markers and IL-6 are mentioned in Table 2. The level of pro-inflammatory cytokine IL6 was found to be significantly elevated (p < 0.001) in T2D + PD, when compared to PD and healthy controls. The levels of bone resorption markers, including serum ALP (P < 0.001), TRAP5b (P < 0.001) and CTx (P < 0.001) were all statistically significant in T2D + PD, when compared to periodontitis and healthy controls. PD subjects also showed an increased expression of bone loss biomarkers when compared with healthy subjects. This indicates inflammation mediated bone loss in PD as well as T2D as both are chronic inflammatory diseases.

**Table 2:** Serum levels of bone resorption markers.

Parameter	Healthy Controls (HC)	PD	T2D + PD
ALP (U/L)	177.42 ± 8.31	209.93 ± 9.14	277.33 ± 6.56∗∗∗
CTx (ng/mL)	16.38 ± 3.70	25.04 ± 2.12	35.59 ± 2.8∗∗∗
TRAP5b (ng/mL)	1.6 ± 0.26	3.23 ± 0.23	5.04 ± 0.53∗∗∗
IL6 (ng/mL)	2.9 ± 1.8	3.1 ± 1.5	4.5 ± 2.1∗∗∗

Values represented as Mean ± SD; ∗∗∗p < 0.001; PD - Periodontitis, T2D + PD -Type 2 diabetes with periodontitis.

## 4 Discussion

Chronic inflammation primarily affects osteoclast differentiation and leads to bone loss.^22^ T2D and periodontitis, as chronic inflammatory diseases, have a profound influence on osteoclastogenesis. Osteoclasts play a key role in alveolar bone loss associated with periodontitis. This study demonstrates some interesting observations with isolated PBMCs from subjects with T2D + PD differentiated into higher number of multinucleated osteoclasts (Fig. 1A) when supplemented with RANKL and MCSF in vitro compared with those cells isolated from healthy control subjects with and without periodontitis. The healthy controls also showed multinucleated osteoclasts that are CD14^+^ positive and TRAP positive as well under the influence of RANKL and MCSF supplementation but they were fewer in number and smaller in size.

CD14^+^ cells are found in peripheral blood, and they differentiated into osteoclasts.^23^^,^^24^ CD14^+^ cells emanating from PBMCs were selected in this study as they exhibit strong expression of RANKL and lack osteoprotegerin (OPG). Earlier studies on priming of osteoclasts from PBMCs in osteopenic diseases have confirmed increased number of TRAP positive cells in affected subjects when compared to healthy controls.^25^^,^^26^ Increased osteoclastic activity in experimental diabetes associated with periodontitis has been reported in mouse models.^27^ However, in this study, for the first time the influence of T2D in increasing priming of osteoclasts is demonstrated in human subjects with and without periodontitis.

IL-6 is a major mediator of immuno inflammatory response in periodontitis,^28^ and its sharp increase in serum in T2D + PD as presented in this study, aligns with the observation that glycemic control may be associated with the inflammatory response in chronic periodontitis that is strongly osteoclastogenic.^29^ IL6 and its downstream mediators such as Janus Kinase 2 (JAK2) and Signal Transducer and activator of transcription 3 (STAT3) also mediate osteoclastogenesis by upregulating RANKL which eventually leads to high-level expression of TRAF6, cFos, NFATc1 and osteoclastic enzyme, cathepsin K.^30^ To substantiate this observation, we have assessed the protein expression levels of the key osteoclast transcription factors in this study.

TRAF6 is an adaptor protein, critical for osteoclast differentiation and for RANKL up-regulation.^31^ The increase in the protein expression of TRAF6 found in T2D + PD, when compared with PD, confirms the transformation of pre-osteoclasts into mature osteoclasts. cFos and NFATc1 are indispensable transcription factors for the differentiation of osteoclasts. Also, NFATc1 is the master regulator of RANKL mediated osteoclast differentiation.^32^ In the present study, NFATc1 protein expression levels were higher in T2D + PD, as shown earlier in studies with periodontitis.^33^

The nuclear translocation of NFATc1 results in expression of osteoclast marker cathepsin K, which is a key lysineprotease secreted by osteoclasts.^34^ The inflammatory cytokine positively activates the osteoclastic expression of cathepsinK, a major link connecting periodontitis and systemic disease such as T2D. A sharp rise in the levels of cathepsin K levels in the gingival crevicular fluid (GCF) of subjects with periodontitis has been reported by Gajendran etal.^35^ CathepsinK has been suggested as a biochemical marker of alveolar bone loss in chronic periodontitis.^36^ In this study we demonstrated an increased cathepsinK expression in T2D + PD conditions as compared to periodontitis alone and healthy controls.

We further studied the influence of T2D and periodontitis on osteoclast induced bone resorption by analysing the serum levels of bone remodelling markers such as ALP, CTx and TRAP5b. A study by Payne confirms that some serum bone resorption markers are not only associated with systemic bone loss but also with alveolar bone loss.^37^

Of the different bone resorption markers studied, serum CTx is a sensitive, predictable, and specific marker that relating directly to osteoclastic bone resorption. Furthermore, CTx is a specific bone turnover marker for T2D and an increase in its levels has been shown to correlate with the duration of diabetes.^38^ In line with the existing literature, the results of this study also demonstrated significant increase in CTx levels in the T2D + PD compared to the PD and suggests that T2D induces higher bone resorption resulting in alveolar bone loss.

TRAP5b is an osteoclast specific enzyme that reflects the number and activity of mature osteoclasts.^39^ Increased TRAP5b levels have also been reported in alveolar bone loss associated with ovariectomy in experimental periodontitis rat models,^40^ and a positive correlation between T2D and TRAP5b levels has been reported.^41^ Currently no notable literature is available relating TRAP5b in periodontitis and T2D. The combination of TRAP5b and CTx, a marker reflecting bone degradation by osteoclast derived cathepsin K, enables a more profound understanding of alveolar bone turnover in our study.

In line with the existing data, the results of this study showing significant rise in CTx levels in T2D + PD compared to PD suggest that T2D induces higher bone resorption resulting in alveolar bone loss. This was also evident clinically among the study subjects as increased clinical attachment loss and probing depth in periodontium.

Alkaline phosphatase (ALP) is a valuable biomarker for determining the severity of periodontal disease progression and alveolar bone loss.^42^ Increased serum ALP levels are an independent risk factor for T2D.^43^ The elevated levels of ALP enzyme found in subjects with T2D + PD in this study indicates destructive processes occurring in the alveolar bone, suggesting advanced periodontal breakdown.

Oral health has a huge impact on the quality of human life and early management of periodontitis is highly essential especially with patients suffering from chronic diabetes.^2^^,^^4^ Type2 diabetes has reached epidemic proportions in India with 77 million suffering from the disease. The total diabetic population across the globe being 463 million^44^ and the prevalence of periodontal disease among diabetics is 67.8%.^45^ T2D associated hyperglycemia always facilitate dysbiosis of oral microbiome. Periodontal pockets with biofilms formed due to microbial lesions results in systemic manifestation, as they release harmful exo and endotoxins into the blood stream influencing inflammation. Effective periodontal treatment improves glycemic control and a reasonable control of diabetes early on could also reduce periodontal changes and certainly prevent tooth loss.^46^ The interdependent pathogenesis of both periodontitis and T2D should be carefully investigated by both oral health providers and physicians to minimize systemic inflammation and bone loss.

In summary, there is a marked increase in osteoclastogenesis and elevated expression of osteoclastic markers, including TRAF6, cFos, NFATc1, and cathepsin K with periodontitis and T2D. Consistent with these observations, serum levels of bone resorption enzymes/markers like TRAP5b, and CTx were also found to be increased in subjects with T2D with PD.

In total, this study addresses a clinically relevant yet underexplored interface between systemic metabolic disease and periodontal bone loss, demonstrating T2D-enhanced osteoclastogenesis using patient-derived PBMCs cultured in vitro, simultaneously substantiated by upstream RANKL signalling markers and systemic bone turnover indices. This multiparametric approach, spanning molecular, cellular and biochemical domains across three clinically well-defined groups, strengthens the mechanistic interpretation of the findings and advances this work beyond cell line studies.

However, the cross-sectional nature of this study and lack of radiographic analysis to evaluate alveolar bone loss are the major limitations of this study. Nevertheless, this observation supports the persistent and continued roles of sustained T2D in mediating alveolar bone loss in periodontitis.

## 5 Conclusion

The major findings of this study highlight the pivotal role of diabetes in aggravating alveolar bone loss in chronic periodontitis at the molecular level. Future longitudinal studies could further strengthen the translational potential of these findings, enabling the identification of specific agents or drugs to inhibit enhanced osteoclast differentiation and bone resorption in patients with both chronic periodontitis and diabetes.

## Ethics approval and consent to participate

The study was conducted in accordance with the Declaration of Helsinki, and the protocol was approved by the Institutional Ethical committee(SBDC - ECM 104/01/15/04)). All procedures performed in studies involving human participants were in accordance with the ethical standards of the institutional and/or national research committee.

## Consent for publication

Not applicable.

## Authors' contributions

RM: Investigation, Methodology, Writing—original draft RP: Investigation, Methodology, Supervision, Writing—original draft ND,BS,BJ: Writing—review & editing.

## Data availability

All data generated or analysed during this study are included in this article. Further inquiries can be directed to the corresponding author.(MR).

## Human ethical statement

The present study was conducted following the ethical standards of the institutional research committee and in accordance with the Declaration of Helsinki. Ethical approval for this study was obtained from the Institutional Ethical Committee (IEC), under the approval number (SBDC - ECM 104/01/15/04).

Written informed consents were obtained according to the Indian Council of Medical Research (ICMR) and World Medical Association Declaration of Helsinki Guidelines. This study was carried out in accordance with the Strengthening the Reporting of OBservational studies in Epidemiology (STROBE) guidelines.

## Funding

This research did not receive any grant from funding agencies in the public, commercial, or not-for-profit sectors.

## Declaration of competing interest

The authors declare that they have no known competing financial interests or personal relationships that could influence the work reported in this paper.
